# Comparative efficacy of 0.1% and 0.15% Sodium Hyaluronate on lipid layer and meibomian glands following cataract surgery: A randomized prospective study

**DOI:** 10.1371/journal.pone.0306253

**Published:** 2024-07-30

**Authors:** Seung Ahn Yang, Mu Ryang Jeong, Cheon Ho Park, Ki Bum Cheon, Jun Ho Chang, Ji Eun Lee

**Affiliations:** 1 Department of Ophthalmology, Pusan National University School of Medicine, Yangsan, South Korea; 2 Department of Ophthalmology, Pusan National University Yangsan Hospital, Yangsan, South Korea; 3 Research Institute for Convergence of Biomedical Science and Technology, Pusan National University Yangsan Hospital, Yangsan, South Korea; 4 Department of Medicine, Pusan National University School of Medicine, Yangsan, South Korea; Democritus University of Thrace, GREECE

## Abstract

**Purpose:**

To compare the efficacy of a 0.15% HA with that of 0.1% HA eye drops for DES after cataract surgery.

**Methods:**

This study was double blinded, randomized and prospective study, and conducted in 69 participants (70 eyes) from Pusan National University Yangsan Hospital and executed from February 1, 2022 to November 30, 2022. Participants were adult cataract patients with normal lid position, not suffering from any other ocular disease and not meet the exclusion cirteria of clinical trial. Participants were randomly divided into two groups: 35 participants (17 males and 18 females) in the 0.1% HA group and 34 participants (19 males and 15 females) in the 0.15% HA group, receiving treatment six times daily for 6 weeks following cataract surgery. Subjective and objective assessments were performed at preoperative and postoperative visits, including ocular surface disease index score, tear break up time, corneal staining score, Schirmer’s I test score, lipid layer thickness), meiboscore, and biochemical analysis of the eye drops.

**Results:**

Throughout the study, the postoperative ocular surface disease index score was significantly lower in the group receiving 0.15% hyaluronic acid than in the group receiving 0.1% hyaluronic acid. Additionally, the postoperative ocular surface disease index score showed a significant positive correlation with the postoperative use of 0.15% hyaluronic acid and the preoperative Schirmer’s I test score. In multivariate analysis, treatment with 0.15% hyaluronic acid and the preoperative ocular surface disease index score were significant independent parameters affecting the postoperative ocular surface disease index score.

**Conclusion:**

The use of 0.15% hyaluronic acid is recommended for its potential advantages in alleviating symptoms following cataract surgery, making it a viable alternative to traditional 0.1% hyaluronic acid treatment.

**Trial registration:**

ISRCTN95830348.

## Introduction

Cataract extraction surgery is a minimally invasive technique, usually performed on an outpatient basis. Patients generally undergo short and uncomplicated recovery periods [1,2] Following surgery, patients are prescribed eye drops that serve to reduce surgery-induced inflammation and promote visual recovery [3,4]. The main objective is to prevent postoperative issues such as macular edema, corneal edema, and endophthalmitis [5,6].

In addition to rare but sight-threatening complications, a significant percentage of patients experience symptoms consistent with dry eye syndrome (DES) after cataract surgery [7]. These symptoms include ocular discomfort, visual disturbances, and tear film instability, which are characteristic of DES—a multifactorial condition affecting the precorneal tear film. DES is characterized by foreign body sensations, ocular soreness, and ocular pain [8,9]. Factors responsible for the development of dry eyes after cataract surgery include the use of antibiotic-steroid eye drops, nerve transection during corneal incisions, and local inflammation, all of which can disrupt tear film stability [4,10].

Recent publications have reported that patients who also receive artificial tears in the postoperative regimen experience significantly less subjective discomfort and show improved tear break-up time (TBUT) scores [11–13]. One such artificial tear medication is sodium hyaluronate, commonly referred to as hyaluronic acid (HA). HA is an anionic glycosaminoglycan with viscoelastic properties that has been widely used as a lubricant in eye drops in recent decades. By effectively retaining water and preventing dehydration, HA enhances lubrication of the ocular surface, stabilizes tear film, promotes epithelial healing, and ameliorates the severity of dry eye symptoms [14,15]. Treatment with HA-only topical drops has also been shown to increase quality of life scores and patient satisfaction, especially in cases of mild-to-moderate DES [16]. This study aimed to compare the efficacy of a 0.15% HA with that of 0.1% HA eye drops for DES after cataract surgery, and this comparison was made by quantitatively evaluating clinical manifestations. Subjective parameters included ocular surface disease index (OSDI) score and objective parameters included TBUT, corneal staining score (CSS), Schirmer’s I test score, lipid layer thickness (LLT), meiboscore, and biochemical analysis of the eye drops [17].

## Materials and methods

This study was a prospective, randomized, double-blinded, and controlled clinical trial aimed at evaluating the efficacy of 0.15% HA in terms of TBUT, CSS, Schirmer’s I score, OSDI score, LLT, meiboscore and biochemical analysis in patients following cataract surgery. The study protocol received approval from the Pusan National University Yangsan Hospital Institutional Review Board (05-2022-084) and was performed in accordance with the principles of the Declaration of Helsinki. All participants provided informed consent by writing to take part in the study. The protocol for this clinical trial and supporting CONSORT checklist are available as (S1 Checklist). The recruitment period for the study spanned from May 11, 2022 to November 30, 2022, with the study conducted between May 6, 2022, and February 9, 2023, at Pusan National University Yangsan Hospital, Yangsan, Korea. The trial was registered with the International Standard Randomized Controlled Trial Number (ISRCTN 18755487).

### Study participants

Participants included in this study were adults who underwent cataract surgery at our center from February 1, 2022 to November 30, 2022, exhibited normal lid position and closure and did not have any ocular diseases. Exclusion criteria included patients who had previously used topical artificial tears, anti-inflammatory agents, antibiotics, or other medications that could affect tear production or stimulate tear secretion within 90 days prior to surgery. Patients with a history of ocular surgery, laser, or systemic treatments that could impact tear secretion, autoimmune diseases, evidence of eye surface disorders observed during slit-lamp, or those using contact lenses were also excluded.

**Fig 1:**
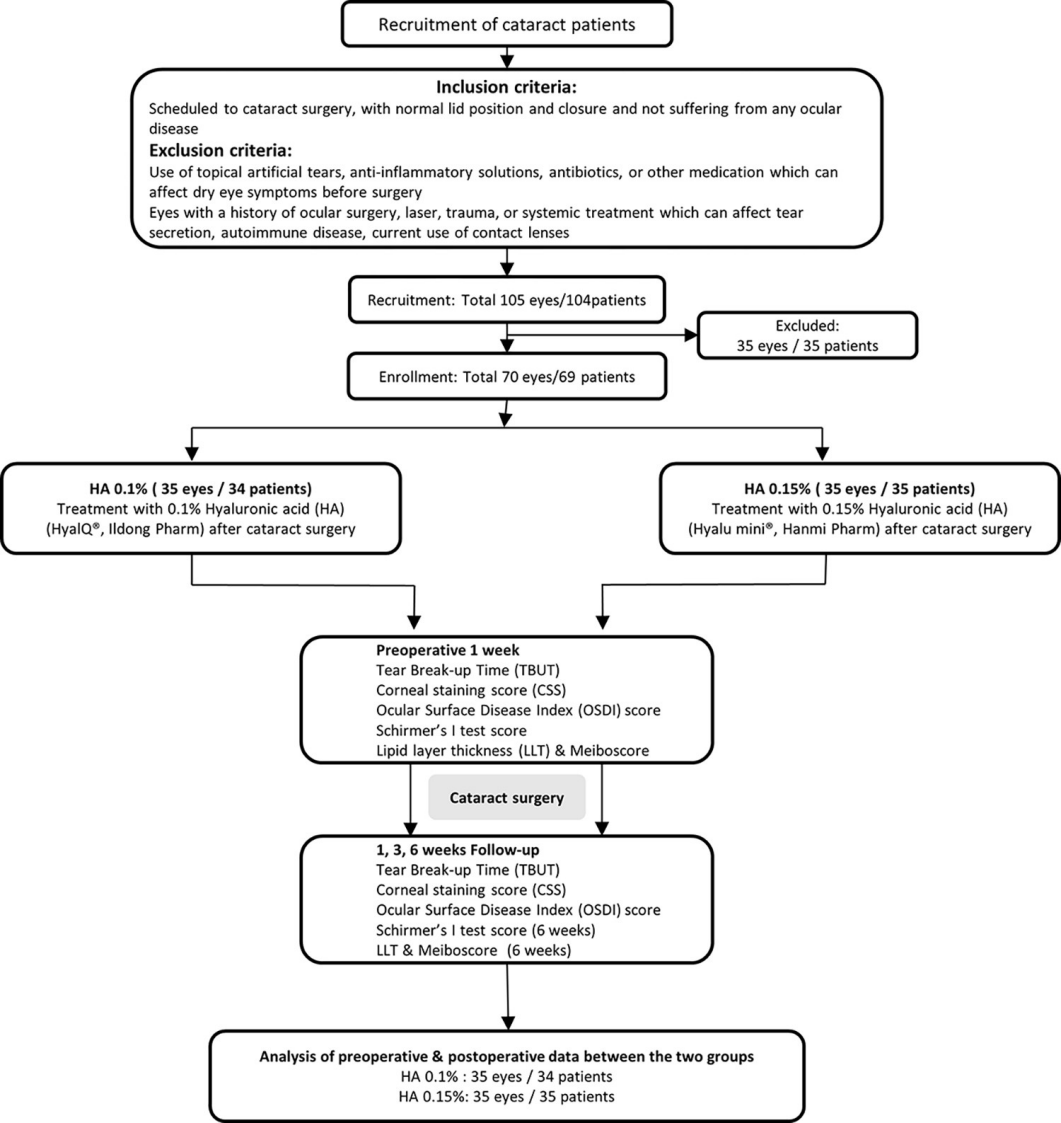
Consort protocol for the study described with flowchart.

The sample size was calculated using MedCalc version 10.0 (MedCalc, Ostend, Mariakerke, Belgium). The minimum sample size requirement for a t-test with an alpha level of 0.05, and a power of 0.8, was calculated to be 21 for each group, and in consideration of 20% dropout rate, 25 patients for each group were needed to recruit. A total of 104 patients (105 eyes) were recruited, and the per-protocol (PP) population consisted of 69 patients (70 eyes) at the Department of Ophthalmology of Pusan National University Yangsan Hospital were enrolled (Fig 1). Eligible subjects were assigned a sequential number with a corresponding randomisation code generated by an independent third party using the SAS version 8.0 (SAS Institute, Inc., Cary, NC). Following the randomization protocol, clinical staff assigned patients to receive either 0.15% HA (Hyalu Mini; Hanmi Pharmaceutical, Inc., Seoul, Korea) or 0.1% HA (HyalQ; Ildong Pharmaceutical, Inc., Seoul, Korea), administered six times daily for 6 weeks following cataract surgery. Clear instructions on how to administer the ophthalmic solutions were provided to the patients by the clinical staff. To maintain blinding of the researchers and participants, medications were dispensed by a pharmacologist, and the specific type of topical medication was not revealed until the completion of the follow-up examination at the end of the study. All patients underwent standard small-incision cataract surgery, which was performed by a single surgeon (JEL). A clear corneal incision 2.8 mm in length was made in the superotemporal region of the eye. Subsequently, all eyes received identical postoperative eye drops consisting of 1.5% levofloxacin, administered four times daily for 2 weeks, and 0.1% fluorometholone, administered four times daily for 1 week, followed a tapering regimen. Additionally, patients received either 0.1% or 0.15% HA six times daily for 6 weeks.

### Clinical measurements

To assess ocular surface status, the following measurements were conducted preoperatively and postoperatively: 1-week preoperative TBUT, CSS, OSDI score, Schirmer’s I test score, LLT, and meiboscore. Following cataract surgery, follow-up visits were conducted at 1, 3, and 6 weeks postoperatively to measure TBUT, CSS, and OSDI score at each visit. Ocular symptoms were evaluated preoperatively and during each follow-up visit using the OSDI questionnaire. LLT was measured using the LipiView Ocular Surface Interferometer (TearScience Inc., Morrisville, NC) to obtain an interferometric image of the tear film, as previously described [18]. Interferometric color units (ICUs) were utilized to measure LLT with one ICU equaling 1 nm of LLT. The recorded measurements included the average LLT obtained from all frame averages, as well as the maximum and minimum LLT. An index C-factor validate the stability of LLT measurements. Participants with interfererometer results showing a C-factor < 0.8 were excluded from the study. LipiView has an upper cut-off value of 100 ICU. The primary outcomes aimed to evaluate changes in TBUT, Schirmer’s I test score, OSDI score, and LLT during the follow-up period between the 0.15% HA group and the 0.1% HA group. The secondary outcome was to determine the baseline factors that affected each clinical parameter at 6 weeks postoperatively. To minimize subjective measurement bias, TBUT, CSS, and OSDI score measurements were conducted by the same surgeon (JEL) at different time points.

The Meibomian gland (MG) images were captured by the same examiner three times under consistent contrast settings, ensuring that the entire MG area was included and clearly visible. The images were evaluated using the Phoenix Meibography imaging module. Although the process was computer-assisted, it necessitated manual tracing of gland boundary. Initially, the observer selected the best image and assessed either the upper or lower eyelid. The lid area and gland boundaries were manually marked, and the area of loss score was automatically calculated, along with a pre-established degree using the Meibomian Scale [19]. Meiboscores were categorized based on the percentage of the lost field, with values ranging from 0 = 0%, 1 ≤ 25%, 2 = 26–50%, 3 = 51–75%, and 4 > 75% [19]. Scores for both upper and lower eyelids were computed, resulting in a total score ranging 0–8 for each participant.

### Statistical analysis

Following the recruiting protocol, we recruited 104 individuals (105 eyes) who met the inclusion criteria. Subsequently, 69 participants (70 eyes) were enrolled based on exclusion criteria and randomized into two groups: 35 participants (17 males and 18 females) in the 0.1% HA group and 34 participants (19 males and 15 females) in the 0.15% HA group. The statistical analysis was then performed on these two groups. All statistical analyses were performed using SPSS for Windows version 26.0 (SPSS Inc., Chicago, IL, USA). Descriptive statistics are reported as mean ± standard deviation. Data normality was assessed using the Kolmogorov–Smirnov test. An independent t-test or chi-square analysis was used to compare the baseline values between the 0.15% HA group and the 0.1% HA group. The time-dependent changes in TBUT, Schirmer’s I test score, OSDI score, and LLT between the two groups were evaluated using repeated-measures analysis of variance (ANOVA). To compare parameters at different time points within each group, ANOVA with a post-hoc paired Tukey’s test was employed. Multiple linear regression analysis was conducted to identify determinant factors associated with the clinical parameters TBUT, Schirmer’s I test score, OSDI score, and LLT at 6 weeks postoperatively. Each variable was initially analyzed using a univariate model, and all significant variables (*p* < 0.10) were subsequently evaluated using a multivariate model with the backward method. The coefficient of determination (R2) in the linear regression was reported, and statistical significance was set at *p* < 0.05.

## Results

A total of 69 participants (70 eyes) were enrolled and randomized into two groups: 35 participants (17 males and 18 females) in the 0.1% HA group and 34 participants (19 males and 15 females) in the 0.15% HA group. Inclusion criteria included patients who completed the study and provided all data at 1, 3, and 6 weeks postoperatively. The clinical and demographic data for both groups are presented in Table 1.

**Table 1:** Preoperative clinical characteristics and demographics.

Parameter	HA 0.1%	HA 0.15%
Age (year)	67.94±9.85	67.57±12.44
Gender (male/female)	17/18	19/15
Laterality (right/left)	20/15	19/16
TBUT (sec)	11.54±4.07	11.23±4.60
Schirmer I test score (mm)	7.49±3.97	8.23±4.74
CSS	0.46±0.111	0.37±0.092
LLT (nm)	67.03±19.18	71.46±16.41
Meiboscore (upper + lower)	3.46±0.80	3.23±0.78
OSDI score	27.33±6.65	28.42±8.18

TBUT: Tear break up time, CSS: Corneal staining score, LLT: Lipid layer thickness, OSDI: Ocular surface disease index.

**Fig 2:**
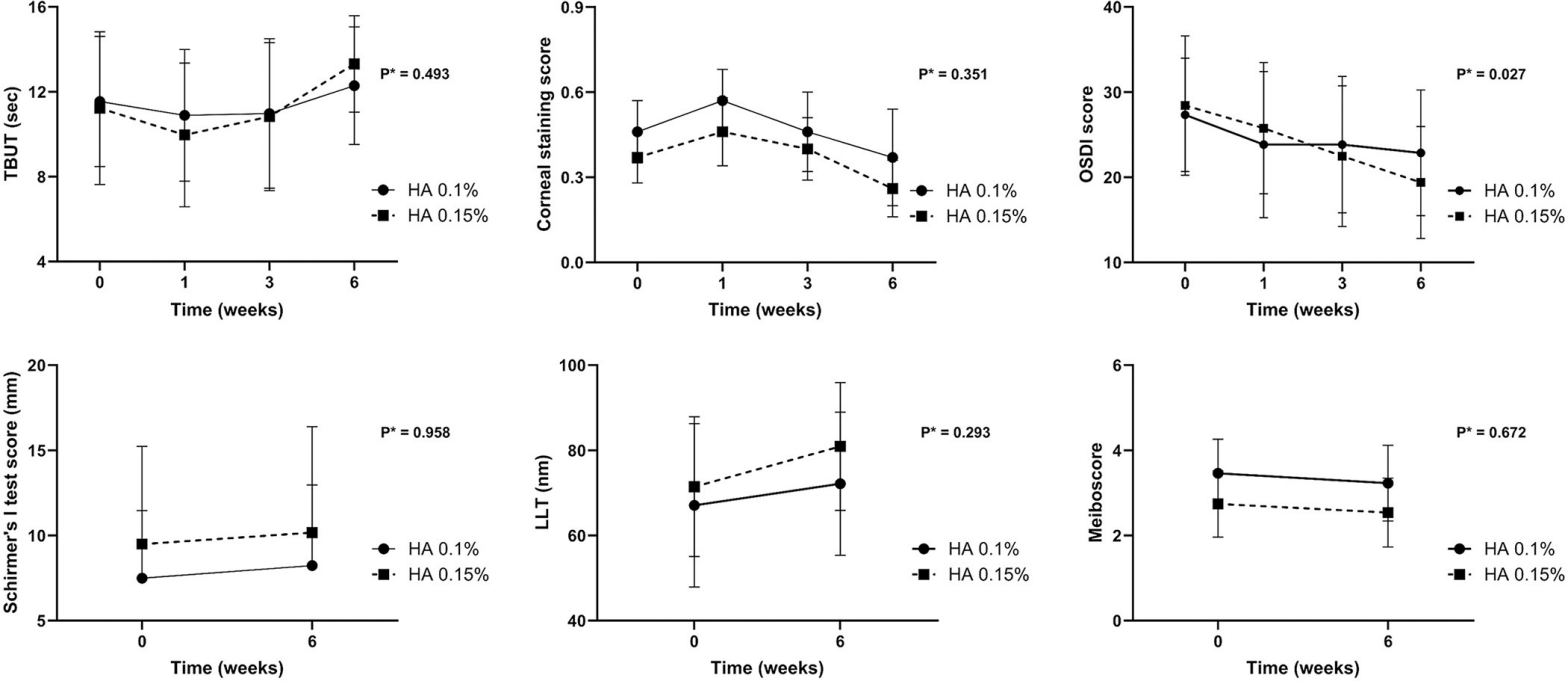
Changes in tear break up time (TBUT), corneal staining score (CSS), ocular surface disease index (OSDI) score, Schirmer’s I test score, lipid layer thickness (LLT), and meiboscore. The OSDI during the follow-up after cataract surgery was significantly different between the participants treated with 0.1% and 0.15% HA (p = 0.027 by repeated measures ANOVA).

As shown in Table 2, the one-way ANOVA test indicated significant improvements in TBUT and OSDI scores in the 0.15% HA group (*p* = 0.001 and <0.001, respectively), as well as LLT improvements in both groups (*p* = 0.037 in 0.1% HA and 0.014 in 0.15% HA) during the follow-up period. Post-hoc Tukey analysis performed for each variable showed that OSDI scores at postoperative 3 and 6 weeks in the 0.15% HA group significantly differed from those at the preoperative visit (*p* = 0.009 and <0.001, respectively, by one-way ANOVA with post-hoc Tukey test). Conversely, all clinical parameters of the 0.1% HA group exhibited no significant differences between the preoperative and postoperative visits. Furthermore, Table 2 and Fig 2 illustrate that the changes in OSDI score from preoperative time point to 6 weeks after cataract surgery were significantly different between the 0.1% and 0.15% HA groups (*p* = 0.027, repeated measures ANOVA).

**Table 2:** Changes in tear break up time (TBUT), corneal staining score (CSS), ocular surface disease index (OSDI) score, Schirmer’s I test score, lipid layer thickness (LLT), and meiboscore in the hyaluronic acid (HA) 0.1% and 0.15% groups.

	Preoperative	Postoperative 1 week	Postoperative 3 weeks	Postoperative 6 weeks	*p* ^a^	*p* ^b^	*p* ^c^	*p* ^d^	*p* value*
						pre vs 1 week	pre vs 3 weeks	pre vs 6 weeks	
TBUT (sec)									
HA 0.1%	11.54±4.07	10.89±4.10	10.97±4.53	12.29±3.77	0.465	0.91	0.938	0.875	0.493
HA 0.15%	11.23±4.60	9.97±4.39	10.83±4.49	13.31±3.27	**0.011**	0.923	0.997	**0.050**	
CSS									
HA 0.1%	0.46±0.11	0.57±0.11	0.44±0.14	0.37±0.17	0.779	0.934	0.958	0.972	0.351
HA 0.15%	0.37±0.09	0.46±0.12	0.40±0.11	0.26±0.10	0.591	0.891	0.988	0.920	
OSDI score									
HA 0.1%	27.33±6.65	23.84±8.57	23.83±10.01	22.87±9.37	0.156	0.342	0.338	0.147	**<0.027**
HA 0.15%	28.42±8.18	25.76±7.71	22.48±8.26	19.38±6.58	**<0.001**	0.477	**0.009**	**<0.001**	
Schirmer’s I test score									
HA 0.1%	7.49±3.97			9.49±5.74	0.094				0.958
HA 0.15%	8.23±4.74			10.17±6.21	0.052				
LLT (nm)									
HA 0.1%	67.03±19.18			72.17±16.81	**0.037**				0.293
HA 0.15%	71.46±16.41			80.91±15.02	**0.014**				
Meiboscore (upper + lower)									
HA 0.1%	3.46±0.80			2.74±0.89	0.083				0.672
HA 0.15%	3.23±0.78			2.54±0.81	0.062				

The OSDI during the follow-up after cataract surgery was significantly different between the participants treated with 0.1% and 0.15% HA (p = 0.027 by repeated measures ANOVA). Statistically significant *p* values are marked in bold (*p* < 0.05).

*p value < 0.05 by repeated-measures analysis of variance.

^a^*p* value < 0.05 one-way analysis followed by post-hoc Tukey analysis.

^b^Between preoperative visit and postoperative 1 weeks.

^c^Between preoperative visit and postoperative 3 weeks.

^d^Between preoperative visit and postoperative 6 weeks.

Correlation coefficients were calculated to evaluate the effects of preoperative clinical measurements on the TBUT, Schirmer’s I test score, OSDI score, CSS, LLT, and meiboscore at 6 weeks postoperatively (Table 3). Notably, Schirmer’s I test score positively correlated with preoperative TBUT and preoperative Schirmer’s I test score (*p* = 0.291 and *p* = 0.654, respectively). OSDI score at postoperative 6 weeks was negatively related to the 0.15% HA group and preoperative Schirmer I test score (*p* = -0.282 and *p* = -0.264, respectively). LLT at postoperative 6 weeks negatively correlated with age (*p* = -0.543) and positively correlated with preoperative LLT (*p* = 0.660). The meiboscore positively correlated with the preoperative meiboscore and negatively correlated with the OSDI score (*p* = 0.740 and *p* = -0.267, respectively).

**Table 3:** Pearson’s correlation coefficients between various demographic and preoperative clinical parameters and clinical measurements at postoperative 6 weeks.

Parameter	At postoperative 6 weeks					
	TBUT	CSS	OSDI	Schirmer’s I test score	LLT	Meiboscore
Age	-0.203	0.177	-0.067	-0.023	**-0.543** **	0.232
Gender (ref:male)	0.221	**-0.290** *	0.091	-0.033	-0.202	-0.102
Group (ref:0.1%)	0.146	-0.07	**-0.282** *	0.058	0.185	0
Preoperative TBUT	-0.093	0.097	0	**0.291** *	-0.165	0.091
Preoperative CSS	0.216	-0.149	0.117	0.004	0.004	-0.18
Preoperative OSDI score	-0.022	-0.125	**0.351** **	-0.088	-0.206	**-0.267** *
Preoperative Schirmer’s I test score	0.212	-0.148	**-0.264** **	**0.654** **	-0.085	0.136
Preoperative LLT	-0.058	0.043	-0.018	0.077	**0.660** **	0.119
Preoperative Meiboscore	-0.111	0.205	-0.064	0.042	0.055	**0.740** **

TBUT: Tear break up time, CSS: Corneal staining score, OSDI: Ocular surface disease index, LLT: Lipid layer thickness.

**p < 0.01 and

*p < 0.05 by Pearson’s correlation analysis.

Multivariate linear regression analysis was performed to examine the influence of independent preoperative parameters on TBUT, CSS, OSDI score Schirmer’s I test score, LLT, and meiboscore at postoperative 6 weeks (Table 4). Preoperative CSS and Schirmer’s I test scores were significant parameters for postoperative TBUT (R^2^ = 0.119, *p* = 0.021 and 0.022, respectively). The 0.15% HA treatment and preoperative OSDI score were significant independent parameters for postoperative OSDI score (R^2^ = 0.121, *p* = 0.018 and 0.024, respectively). Furthermore, age was a significant independent parameter for postoperative LLT (R^2^ = 0.184, *p* < 0.001), and preoperative OSDI and meiboscore were significant parameters for the postoperative meiboscore after 6 weeks (R^2^ = 0.563, *p* = 0.128, and <0.001, respectively).

**Table 4:** Multiple regression analysis to determine the influence of independent preoperative parameters on each clinical measurement at postoperative 6 weeks.

Parameter	At postoperative 6 weeks											
	TBUT		CSC		OSDI		Schirmer’s I test		LLT		Meiboscore	
	B (95% CI)	*p* value	B (95% CI)	*p* value	B (95% CI)	*p* value	B (95% CI)	*p* value	B (95% CI)	*p* value	B (95% CI)	*p* value
Age									-0.543(-1.592, -0.724)	**<0.001**		
Gender (reference: male)			-0.475(-0.856, -0.095)	0.015								
Group (reference: HA 0.1%)					-0.281(-0.02, -0.002)	**0.018**						
Preoperative TBUT							0.396(0.073, 0.719)	0.017				
Preoperative CSS	1.639(0.256, 3.022)	**0.021**										
Preoperative OSDI score					0.349(0.144, 0.679)	**0.024**					-0.013(-0.030, 0.004)	**0.128**
Preoperative Schirmer I test score	0.224(0.033, 0.415)	**0.022**										
Preoperative LLT							0.012(-0.066, 0.090)	0.753				
Preoperative Meiboscore											0.746(0.585, 0.906)	**<0.001**

TBUT: Tear break up time, CSS: Corneal staining score, OSDI: Ocular surface disease index, LLT: Lipid layer thickness, HA:Hyaluronic acid Results of the backward method in the multivariate analysis.

Chemical analysis revealed the concentrations of Na^+^, K^+^, Cl^-^, pH, and osmolality of each drug. The concentration of Na^+^ was higher in the 1.5% HA group, exceeding the normal range. K^+^ was higher in 0.1% HA group, and Cl^−^in both solutions exceeded the normal range. The pH measured at 5.5, which falls below the normal range, whereas the osmolality of both solutions remained within the normal range (Table 5).

**Table 5:** Electrolyte compositions, pH values, and osmolarities of the two drugs tested.

Parameter	Na^+^ (mEq/L)	K^+^ (mEq/L)	Cl^-^ (mEq/L)	pH	Osmolarity (mOsm/kg)
HA 0.1%	127	19.8	142	5.5	283
HA 0.15%	153	<1.0	156	5.5	299

HA:Hyaluronic acid.

## Discussion

The aim of this randomized clinical trial was to compare the effects of 0.15% HA with those of 0.1% HA on the ocular surface following cataract surgery. Among participants who had undergone cataract surgery, 0.15% HA significantly improved the OSDI score at 6 weeks postoperatively compared with 0.1% HA. Furthermore, postoperative treatment with 0.15% HA, as opposed to 0.1% HA, showed a significant positive correlation with the OSDI score at 6 weeks after surgery. Preoperative OSDI values and Schirmer’s I test scores also had a positive impact on the OSDI score at 6 weeks postoperatively.

Despite advances of surgical techniques in cataract surgery, most post-cataract patients still experience dry eye symptoms that vary in severity and duration [3,20]. Corneal nerve transection, prolonged microscope light exposure, use of an aspirating speculum, and heat from phacoemulsification devices are possible risk factors for postoperative dry eye [6,10]. Artificial tears are first-line treatment for dry eye symptoms after cataract surgery [4]. HA stimulates ocular surface tissue healing by humidifying the eye surface and restoring integrity of the corneal and conjunctival epithelium [14]. Recently, artificial tear preparations with increased HA concentrations are introduced, and to our knowledge, no comparative clinical trials have been published to confirm the potential additional benefit of DES after cataract surgery with 0.15% HA over the standard 0.1% HA. Exploring the potential superiority of 0.15% HA in patients who underwent cataract surgery was the primary objective of our study.

Evaluating treatment success in DES is a major challenge because of the weak correlation between signs and symptoms in DES, and the high variability in objective signs [21]. Therefore, the assessment of treatment efficacy using subjective symptoms and objective signs is particularly important in patients with dry eye. TBUT is a surrogate measure of tear film stability. The improvements in TBUT with the use of HA suggest improved integrity of the tear film, which can prevent evaporation and hyperosmolarity owing to its rheological and water-retention properties [13]. Although TBUT increased with the postoperative use of both 0.1% and 0.15% HA, significant improvement was only found in 0.15% HA group in this study. CSS is a valuable clinical tool for assessing epithelial cell viability [22]. While CSS is subjective and observer-dependent, it provides useful information for assessing disease severity and monitoring treatment response [23]. Although the changes in CSS after surgery between the two groups were not significantly different, the CSS of both groups increased at postoperative week 1 and subsequently decreased throughout the follow-up period. This indicates that corneal damage after cataract surgery gradually healed to its preoperative state starting 1 week after surgery.

In addition to its benefits on objective measures, 0.15% HA was also effective for subjective outcomes, showing improvement in symptoms in OSDI score. The OSDI is a unique instrument that assesses the frequency of dry eye symptoms and their impact on vision-related functioning. Because the OSDI score has good to excellent reliability, validity, sensitivity, and specificity, it can be used as a valuable complement to other clinical and subjective measures of dry eye disease by providing a quantifiable assessment of dry eye. It also has the necessary psychometric properties for use as an endpoint in clinical trials of dry eye disease [24,25]. In this study, we used OSDI score to evaluate subjective changes in ocular symptoms after cataract surgery; the 0.15% HA group demonstrated significantly favorable results compared with 0.1% HA, and postoperative OSDI which demonstrated meaningful positive correlation with preoperative OSDI and Schirmer’s I test scores. This suggests that 0.15% HA has superiority from an ocular comfort perspective and that preoperative conditions, such as OSDI and Schirmer’s I test score, could play an important role in alleviating postoperative dry eye symptoms.

Cataract surgery also appears to influence MG function [3,26]. In this study, LLT was significantly improved in both 0.1% and 0.15% HA groups, and postoperative LLT showed significant positive correlation with preoperative LLT as well as young age. By the way, there were no statistically significant differences in meiboscores between 0.1% and 0.15% HA groups. Our previous reports comparing HA with cyclosporine or diquafosol have already shown that better LLT at the preoperative visit could indicate improved postoperative LLT [7,18,26].

Patagiota N, et al. compared the efficacy of 0.1% and 0.2% HA; 0.2% HA showed significant improvements in TBUT at 6 weeks after cataract surgery, indicating better tear film stability. They also used surface discomfort index (SDI) scores to quantify the overall subjective discomfort experienced by post-cataract surgery patients, which also showed significantly superior results with 0.2% HA. When comparing the two concentrations of HA, the 0.2% group demonstrated particularly better scores in the stinging sensation and foreign body sensation indices [11]. Ishioka reported immediate visual impairment following instillation of the 0.3% HA compared with 0.1% HA [27]. Although this blurring of vision is usually temporary, it can cause ocular discomfort associated with OSDI scores. Park et al. compared the efficacy of the 0.1%, 0.15%, and 0.3% HA, and all the groups showed a significant improvement in OSDI scores at 6 weeks, with 0.15% HA showing the most significant improvement [12]. The reason behind the less favorable outcome with the 0.3% concentration might be due to the higher HA concentration, causing increased viscosity, inducing blurry vision, or potentially generating higher-order aberrations that could affect ocular sensation [12,27] Similarly, in the present study, 0.15% HA showed a significantly better OSDI score than 0.1% HA at postoperative 6 weeks. These findings suggest that the 0.15% concentration of HA has fewer potential side effects due to the viscosity-related blurry vision and an advantage in symptom relief as the concentration increases. In other words, 0.15% HA may be more effective in reducing symptoms in patients with dry eye or ocular surface discomfort after cataract surgery.

The electrolyte content, pH, osmolarity, and viscosity of commercially available topical ocular solutions may induce ocular surface damage when used for a long period or overdose [28]. Our study aimed to investigate the differences in subjective symptoms and ocular sensations experienced by patients through a biochemical analysis of the two drugs. Based on our results, the sodium concentration in the 0.15% HA group was slightly higher, whereas in the 0.1% HA group, it was lower than ideal range (142~152.7 mEq/L) [29]. K^+^ concentrations in the 0.1% HA group was higher than ideal range which has been reported to be 4.3–4.6 mEq/L for extracellular fluid. Chloride concentration in both groups were higher than ideal range (104.0~117.4 mEq/L), osmolarity was in the ideal range (260~320 mOsm/kg), and the pH values were both 5.5 which lay below the ideal range (7.0–7.7) [29,30]. Based our previous report which compares the electrolyte component between different concentration of HA, the difference of electrolyte concentration which implies values above and below the ideal range are unlikely to affect the ocular surface. It also reported that osmolarity imbalance can damage cells that hypertonic stress induced human corneal epithelial cell shrinkage and apoptosis in cell culture models, and low pH could increase corneal epithelial permeability. Both 0.1% and 0.15% HA showed osmotic pressure in the normal range and pH was lower than the normal range, so it is thought that both products might have higher corneal permeability [17].

Although this study had the advantage of a double-blind, randomized, prospective assessment designed to minimize bias, it also had some limitations. First, the study duration was relatively short, which may be insufficient for evaluating the long-term impact of different HA concentrations on DES. Second, it is important to note that besides the eye drops used in this study, other products with the same concentration may have slight variations in composition depending on their manufacturers. These differences mean that the results obtained in this study may not be replicable across all concentrations of the same drug. Consequently, even if a different product contains the same active ingredient at the same concentration, the overall formulation and other inactive ingredients may differ slightly, potentially affecting the bioavailability, efficacy, and side effects of the drug.

In conclusion, the present study demonstrated that the postoperative OSDI score was significantly better in the 0.15% HA group than in the 0.1% HA group. Furthermore, there were significant positive correlations between postoperative use of 0.15% HA, preoperative OSDI scores, and Schirmer’s I test scores. Therefore, it is suggested that using 0.15% HA offers advantages in relieving symptoms after cataract surgery and can be considered as an alternative option to conventional 0.1% HA treatment.

## Supporting information

S1 Checklist CONSORT 2010 checklist of information to include when reporting a randomised trial*. (DOC)

S1 File (DOCX)

S2 File (DOCX)
