# Correction: Comparative efficacy of 0.1% and 0.15% Sodium Hyaluronate on lipid layer and meibomian glands following cataract surgery: A randomized prospective study

**DOI:** 10.1371/journal.pone.0317059

**Published:** 2024-12-31

**Authors:** Seung Ahn Yang, Mu Ryang Jeong, Cheon Ho Park, Ki Bum Cheon, Jun Ho Chang, Ji Eun Lee

The following information is missing from the Acknowledgements section: The authors would like to thank the Hanmi Pharmacy for their assistance.
