# Supplementary material for: Comparative efficacy of 0.1% and 0.15% Sodium Hyaluronate on lipid layer and meibomian glands following cataract surgery: A randomized prospective study
Source: PLoS One. 2024 Jul 30;19(7):e0306253. doi: 10.1371/journal.pone.0306253 (PMC11288441; doi:10.1371/journal.pone.0306253)
Supplement: S2 File — (DOCX) [file pone.0306253.s003.docx]

| Title | A randomized double-masked study of topical hyaluronic acid 0.15% and 0.1% in the management of tear lipid layer and meibomian gland in the cataract surgery subjects |
| --- | --- |
| Study design | Single center, double blind, Prospective, Comparative Study |
| Participants | Patients scheduled for cataract surgery with no evidence of dry eyes. |
| Cases | A total of 70 cases. |
| Objective | This study is a single center, double blind, prospective and comparative study which aims to determine the effects of 0.15% hyaluronic acid eye drops on tear film lipid layers and meibomian glands after cataract surgery, compared to those of 0.1% hyaluronic acid eye drops, by collecting and analyzing data obtained during clinical practice. |
| Study drug | - Hyaluronic acid eye drops (0.15%) –Hyalumini ^®^SD - Hyaluronic acid eye drops(0.1%)–HyalQ ^®^SD |
| Criteria for inclusion | In order to be eligible for participation in this study, the subject must:   1. Be male and female aged 19 years or older 2. Have a tear breakup time (TBUT) of 10 seconds or more 3. Have a Schirmer’s I test of 10mm or more 4. Not use hyaluronic acid eyed drops, cyclosporine eye drops, diquafosol-based artificial drops, steroid eye drops or antibiotic eye drops within three months prior to participation 5. Show normal blinking during a slit lamp exam 6. Voluntarily agree to participate in this study |
| Criteria for exclusion | The subject was excluded from the study if the subject:   1. Has Sjögren's syndrome, Stevens-Johnsons-syndrome, or other systemic disease which could affect the condition of dry eye syndrome. 2. Has severe blepharitis 3. Had ocular surgery or laser eye surgery 4. Has severe ocular inflammation/infection 5. Is using eye drops besides treatment for dry eyes, such as glaucoma or allergies. 6. Shows sensitivity to study drugs. 7. Is considered to be ineligible for participation other than the aforementioned exclusion criteria based on the judgement of the principal researcher |
| Prohibited  Concomitant  Medications | Artificial tear drops or topical medications for treatment of dry eyes other than study drugs. |
| Observation Group | - Experimental group: Hyaluronic acid eye drops (0.15%) –Hyalumini ^®^SD - Control group: Hyaluronic acid eye drops(0.1%)–HyalQ ^®^SD |
| Observation period | For more than 3 months |
| Study methods | 1. Double-blind, prospective study 2. Obtain voluntary consent from patients who are scheduled to undergo cataract surgery and without evidence of dry eyes. 3. Visit periods   Preoperative visit : assess baseline characteristics (first visit)  🡪1 week after surgery(second visit)  🡪3 weeks after surgery (third visit)  🡪6 weeks after surgery (final, fourth visit) |

| Assessment variables for efficacy and safety | 1. Primary efficacy variables  - Comparisons of **Tear Breakup Time(TBUT), Corneal staining score (CSS), Ocular surface disease index (OSDI) score, Schirmer’s I test score, Lipid layer thickness (LLT), Meiboscore** changes at last visit from baseline between control and experimental group   ***Tear Breakup Time(TBUT) measurements***:  Add 0.4M Fluorescein paper (Haag-Streit, Switzerland) to the conjunctival sac and allow the patient to blink several times. Measure the time until the appearance of the first dry spot in the dyed tear film under cobalt blue light. Calculate the average value after measuring three times.  ***Corneal staining score(CSS) measurements.***  Add 0.4M Fluorescein paper (Haag-Streit, Switzerland) to the conjunctival sac and allow the patient to blink several times. Evaluate the staining of all five regions of the cornea through slit-lamp examination and measure the corneal staining score by modified National Eye Institute grading scale.  ***Ocular surface disease index (OSDI) score measurements***:  The OSDI score assesses a total of 12 items related to dry eyes. Subjects rate their responses on a 0 to 4 scale with 0 corresponding to “none of the time” and 4 corresponding to “all of the time.” A final score is calculated ranging from 0 to 100, with scores 0 to 12 representing normal, 13 to 22 representing mild dry eye disease, 23 to 32 representing moderate dry eye disease, and greater than 33 representing severe dry eye disease.  ***Schirmer’s I test measurements***:  Add anesthetic eye drops (Alcaine^®^, Alcon, Ft Worth, TX, USA) 5 minutes before placement of a paper strip on the lateral 1:2 point of the lower eyelid. Measure the length of the wet part of the paper strip after 5 minutes of placement.  ***Lipid layer thickness (LLT) and Meiboscore measurements***:  LipiView^®^ ocular surface interferometer (TearScience^®^Inc, Morrisville, NC, USA) measures the thickness of the lipid layer in nanometers and visualizes meibomian gland structure by assessing optical interference patterns produced by reflected light from the tear film lipid layer. Use of eye drops containing lipid substances was prohibited prior to testing. The patient was stabilized for 30 minutes prior to testing in order to minimize effects due to eyelids. During the observation period, we measured the average thickness of the tear film lipid layer and analyzed the image of meibomian glands obtained by LipiView^®^. Grades were assigned by the degree of atrophy of meibomian glands in upper and lower eyelids. 0 points were assigned if there was no atrophy, 1 point if atrophy was less than one thirds, 2 points if between one and two thirds, 3 points if over two thirds.   1. **Secondary efficacy variables**  - Comparison of changes in **TBUT, CSS, OSDI score, Schirmer’s I test score, LLT, and meiboscore** during follow-up period between control and experimental group   Efficacy variables and corresponding method of assessment are identical to primary efficacy variables.   - At final visit, an analysis of baseline factors that affect parameters was made in each group   Efficacy variables and corresponding method of assessment are identical to primary efficacy variables.   - Analysis of baseline factors affecting the amount of changes in parameters from baseline to last visit in each group was made.   Efficacy variables and corresponding method of assessment are identical to primary efficacy variables.   1. Safety variables  - Adverse drug reactions |
| --- | --- |
| Study Flow | \| Visit \| Baseline \| 1 month  ±1week \| 3 months  ± 1week \| Final visit \| \| --- \| --- \| --- \| --- \| --- \| \| Subject consent \| O \|  \|  \|  \| \| Basic information of subject \| O \|  \|  \|  \| \| Medical/Surgical History \| O \|  \|  \|  \| \| Concomitant Medications \| O \|  \|  \|  \| \| Inclusion/exclusion criteria \| O \|  \|  \|  \| \| TBUT \| O \| O \| O \| O \| \| CSS \| O \| O \| O \| O \| \| OSDI score \| O \| O \| O \| O \| \| Schirmer’s I test \| O \|  \|  \| O \| \| LLT \| O \|  \|  \| O \| \| Meiboscore \| O \|  \|  \| O \| \| Adverse drug reactions \|  \| O \| O \| O \| |
| Statistical analysis of efficacy and safety assessment variables | 1. Adjusted covariance and normality verification  When demographic variance is not equal between groups, ANCOVA is used to integrate unmatched variants to covariants, and normality test is done with Komogorov-Smimov analysis.  2. Primary efficacy variables   - Comparisons of **TBUT, CSS, OSDI score Schirmer’s I test score, LLT, Meiboscore,** changes from baseline to last visit between control and experimental group   ANOVA with a post-hoc paired Tukey’s test is used to compare TBUT, CSS, OSDI score Schirmer’s I test score, LLT, Meiboscore changes of the final visit from baseline. At the final visit, repeated measure of ANOVA is used to compare efficacy variables in time-depnedent changes between two groups.  3. Secondary efficacy variables   - Comparisons of **TBUT, CSS, OSDI score Schirmer’s I test score, LLT, Meiboscore**  during the follow-up period between control and experimental groups. - In the final visit, an analysis of factors that affect postoperative TBUT, CSS, OSDI score Schirmer’s I test score, LLT, Meiboscore was done by multiple regression analysis.  1. Safety variables  - Adverse drug reactions   Results related to adverse reactions are presented, including the number of subjects with adverse reactions, incidence rates of adverse reactions, and cases of adverse reactions. Pearson’s Chi-square test or Fisher’s Exact test is used to analyze the difference between groups. |
| Experimental period | 12 months from IRB approval |
